# Emergency Surgery Score as an Effective Risk Stratification Tool for Patients Undergoing Emergency Surgeries: A Narrative Review

**DOI:** 10.7759/cureus.26226

**Published:** 2022-06-23

**Authors:** Pravin Saxena, Abhijit Nair

**Affiliations:** 1 Department of Cardiac Anesthesia, National Heart Center, Royal Hospital, Muscat, OMN; 2 Anesthesiology, Ibra Hospital, Ibra, OMN

**Keywords:** postoperative complications, morbidity and mortality, emergency laparotomy, emergency surgery, emergency surgery score

## Abstract

Several risk stratification tools have been described for quantifying perioperative morbidity, mortality, and adverse events in patients undergoing elective and emergency surgeries. These tools help in decision-making, determining the prognosis and communicating it with patients and family members, and planning admissions to the intensive care units (ICU) if necessary. Emergency surgery poses quite a unique challenge in terms of deranged physiology, age, and comorbid conditions, and often carries a higher incidence of morbidity and mortality. Very few risk stratification tools are available to reliably predict the risk posed by emergency surgical interventions. One of the recently described tools is the Emergency Surgery Score (ESS), which comprises three demographic variables, 10 comorbidities, and nine laboratory variables, the scores of which add up to 29. Several studies have demonstrated that ESS reliably predicts morbidity, mortality, and the need for ICU admission, predicting infectious complications like pneumonia and renal failure. In this review, we analyze the current literature to investigate the efficacy and reliability of ESS as a risk stratification tool for patients undergoing emergency surgeries.

## Introduction and background

There are several validated scoring systems currently available to stratify risk in patients undergoing surgeries. These scoring tools are commonly referred to as risk stratification tools. The most commonly used among these are the American Society of Anesthesiologists’ physical status (ASA-PS) classification system, Physiological and Operative Severity Score for the Enumeration of Mortality and Morbidity (POSSUM), Portsmouth-POSSUM (P-POSSUM), Acute Physiology and Chronic Health Evaluation (APACHE), Surgical Outcome Risk Tool (SORT), Surgical Apgar Score (SAS), Charlson Comorbidity Index (CCI), and Perioperative Mortality Risk Score (PMRS) [1]. Although ASA-PS is the most commonly used risk prediction tool in patients undergoing surgery, it is not consistent with predicting risk for emergency surgeries [2].

APACHE II and P-POSSUM are very popular risk stratification tools used for predicting mortality after intensive care unit (ICU) admission. APACHE II score is derived from 12 routine physiological measurements taken during the first 24 hours after admission, age, and previous medical issues. The APACHE II scoring has been reported to be better suited for medical ICU patients than emergency surgical patients [3]. Hansted et al. performed a post-hoc analysis of 885 patients undergoing emergency abdominal surgery and concluded that the APACHE II score predicted mortality moderately and admission to the ICU poorly in these patients [4]. Although POSSUM and P-POSSUM have been validated for predicting risk in patients undergoing emergency and elective surgeries, they cannot be used for trauma patients and patients undergoing surgeries following trauma [5,6].

Gawande et al. described and validated SAS, which is based on the lowest heart rate, lowest mean arterial pressure, and blood loss, in the year 2007 for general and vascular surgeries [7]. It is a simple 10-point scoring system performed intraoperatively that alerts the anesthesiologist and the surgeon regarding adverse postoperative events in patients and thus warrants meticulous monitoring. A higher score was suggestive of better outcomes at 30 days. Subsequently, SAS has been applied and validated for all surgical specialties [8]. SAS cannot predict mortality or morbidity before surgical intervention.

SORT is a preoperative risk prediction tool developed for predicting death within 30 days of surgery in adults undergoing non-cardiac, non-neurologic surgeries. The variables involved are age, ASA-PS grade (III, IV, and V), the urgency of surgery (expedited, urgent, immediate), specialty, the severity of the surgery, and the presence of cancer [9]. Although the SORT score considers the nature of the surgery, i.e., emergency, it does not take into account any biochemical derangements due to the acute deterioration warranting an emergency surgical intervention. In a study by Oakland et al. involving 3,305 patients undergoing abdominal surgery, SORT underpredicted adverse outcomes in the higher-risk group [10].

Bilimoria et al. developed the American College of Surgeons National Surgical Quality Improvement Program (ACS-NSQIP) Surgical Risk Calculator based on reliable multi-institutional clinical data to estimate the risks of most operations and allow clinicians to make decisions using empirically derived, patient-specific postoperative risks [11]. Alzahrani et al. validated the ACS-NSQIP Surgical Risk Calculator for patients undergoing gastrectomy for stomach carcinoma and found that it had a low predictive ability for perioperative adverse events, and therefore further research and changes in the tool were needed [12]. The search for a robust and comprehensive risk stratification tool continues with a view to helping clinicians predict adverse perioperative events in patients undergoing emergency surgical interventions.

The purpose of this narrative review is to investigate the efficacy and reliability of the Emergency Surgery Score (ESS) as a risk stratification tool for patients undergoing emergency surgeries by analyzing recently published articles. The other objectives are to examine the utility of ESS in patient counseling, prognostication of family members, and benchmarking the quality of emergency surgical interventions.

## Review

Sangji et al. developed and validated a novel scoring tool, the Emergency Surgery Acuity Score (ESAS) to predict mortality, identify patients who require close postoperative monitoring, and for risk stratification in patients undergoing emergency surgeries [13]. This was derived from the data of 18,349 patients retrieved from the ACS-NSQIP database. ESAS, also referred to as the Emergency Surgery Score (ESS), comprises three demographic variables, 10 comorbidities, and nine laboratory variables. The total score adds up to 29 (Table 1). The 30-day complications gradually increase from 7% to 53% to 91% with scores of 0, 7, and 15, respectively. It has been estimated that with an ESS of more than 15, the complication rate observed was up to 92%.

**Table 1:** Variables included in the ESS scoring system and the points allocated COPD: chronic obstructive pulmonary disease; ESS: Emergency Surgery Score; ED: emergency department; INR: international normalized ratio; SGOT: serum glutamic oxaloacetic transaminase; WBC: white blood cells count

Variables	Points allocated
Demography	
Age more than 60 years	2
White race	1
Any transfer from outside ED or acute care hospital	1
Any transfer from an acute care hospital inpatient facility	1
Comorbidities	
Ascites	1
Body mass index less than 20 kg/m^2^	1
History of COPD	1
Disseminated cancer	3
Dyspnoea	1
Functional dependence	1
Hypertension	1
Steroid use	1
Ventilator requirement 48 hours preoperatively	3
More than 10% weight loss in last 6 months	1
Laboratory	
Albumin less than 3.0 U/L	1
Alkaline phosphatase more than 125 U/L	1
Blood urea nitrogen more than 40 mg/dL	1
Creatinine of more than 1.2 mg/dL	2
INR more than 1.5	1
Platelets less than 150 x 10^3^/µL	1
SGOT more than 40 U/L	1
Serum sodium more than 145 mmol/dL	1
WBC less than 4.5 x 10^3^/µL or 15-25 x 10^3^/µL	1
WBC more than 25 x 10^3^/µL	2

Later in 2017, Sangji et al. described and validated the Physiological Emergency Surgery Acuity Score (PESAS), which assessed the severity of disease at presentation in patients undergoing emergency surgery [14]. A 15-point score was derived from 24,702 patients undergoing emergency surgery. PESAS took into account the physiological derangements and the seriousness of the patient’s disease before surgery and hence was more refined than ESAS.

Subsequently, many researchers have investigated the reliability of ESS by analyzing patient data from various national databases and also by prospectively following up on patients undergoing emergency surgeries. The surgeries that have been included are emergency general surgeries (EGS) like laparotomy for perforation peritonitis, appendicitis, intestinal obstruction, and obstructed hernias. We searched databases such as PubMed, Google Scholar, Web of Science, ScienceDirect, and Scopus by using the following keywords: "Emergency Surgery Score, Emergency Surgery, Emergency Laparotomy, Morbidity, Mortality, and Postoperative Complications." All articles were carefully screened and the relevant articles that used ESS were selected and reviewed. A summary of the relevant articles is depicted in Table 2 [13-23].

**Table 2:** Table summarizing the studies in which ESS was used as a risk stratification tool ESS: Emergency Surgery Score; EGS: emergency general surgery; PESAS: Physiological Emergency Surgery Acuity Score

S. no.	Author/year	Number of patients	Conclusion
1	Sangji et al./2016	18,349	Validation of ESS done for patient counseling, need for monitoring, and benchmarking quality of emergency surgeries undertaken
2	Sangji et al./2017	24,702	Derived 15-point-score PESAS that took into consideration physiological derangements and seriousness of patient’s condition
3	Nandan et al./2017	37,999	ESS predicted 30-day complications following emergency surgeries and can be used for prognostication and for benchmarking the quality of emergency surgeries
4	Han et al./2018	90,412	Predicted postoperative complications like surgical site infections, sepsis, and pneumonia; can be used for preoperative prognostication and benchmarking the quality of emergency surgeries
5	Gaitanidis et al./2020	124,335	ESS predicted morbidity and morbidity in elderly patients undergoing emergency surgeries
6	Kaafarani et al./2020	1,649	Used effectively for patient/family counseling, patient triage, and benchmarking the quality of surgeries
7	El Hechi et al./2021	1,347	ESS accurately predicted the need for respiratory and renal support in the postoperative period
8	El Hechi et al./2021	715	ESS predicted mortality, mortality, and need for ICU admission, especially for patients aged between 65-74 years
9	AlSowaiegh et al./2021	84,694	ESS accurately predicted EGS patients requiring discharge to rehabilitation or nursing facilities; helpful for preoperatively counseling patients/families and improving the chances of early discharge
10	Naar et al./2021	359,849	ESS predicted outcomes even with missing data; was effective for counseling patients/family members and as a benchmark for quality of emergency care
11	Christou et al./2022	214	ESS was validated in Greek patients undergoing emergency surgeries

Nandan et al. investigated the ability of ESS to predict the occurrence of 30-day postoperative complications after emergency surgeries [15]. The 30-day complications considered were surgical site infections, acute respiratory failure, and renal failure. The authors found that of the 37,999 patients who were screened, 14,446 patients (38%) developed at least one 30-day complication. They concluded that ESS can be used to predict 30-day complications following emergency surgeries and can also be used for family prognostication and as a benchmark for assessing quality following emergency surgeries.

Han et al. used ESS to investigate the occurrence of postoperative infectious complications in patients undergoing EGS, for preoperative clinical decision-making, and as quality benchmarking of infection rates for such surgeries [16]. The authors analyzed the data derived from the 90,412 patients who fulfilled the inclusion criteria. On analysis, the authors found that ESS reliably predicted postoperative infectious complications (surgical site infections, sepsis/septic shock, pneumonia). They suggested that ESS could be used for preoperative clinical decision-making and quality benchmarking of infection rates after emergency general surgery.

Gaitanidis et al. investigated the predictive accuracy of ESS in elderly patients including octogenarians and nonagenarians undergoing EGS [17]. On analysis, the authors concluded that although ESS could accurately predict postoperative outcomes in elderly patients, the predictive power was not very accurate when applied to octogenarians and nonagenarians. They included 124,335 patients, out of which 34,215 (28%) were octogenarians and 7,239 (6%) were nonagenarians. On analysis, the authors found that ESS accurately predicted mortality (AUC 0.81) in patients aged more than 65 years. In octogenarians and nonagenarians, the mortality prediction was moderately well (AUC 0.77 and 0.69 respectively). They concluded that ESS accurately predicts mortality and morbidity in the elderly EGS patients, but its accuracy in predicting morbidity among nonagenarians was lower.

Kaafarani et al. conducted a prospective, multicenter study involving 1,649 patients to validate ESS in high-risk non-trauma emergency laparotomy patients [18]. On analysis, the authors concluded that ESS could be reliably used for perioperative patient and family counseling, triaging patients to the ICU, and benchmarking the quality of emergency general surgery care. Although the ESS was developed from a patient database in the United States, Christou et al. conducted a study to validate ESS in Greek patients undergoing emergency surgery [19]. On analysis, the authors concluded that ESS and the surgical outcomes were statistically significant in both Greek and American patients undergoing emergency laparotomy. Therefore, the score can be considered for patients from all over the world irrespective of their ethnicity.

El Hechi et al. conducted a post-hoc analysis of a 19-center prospective observational study (EAST multicenter study) of adult patients undergoing emergency laparotomies [20]. The study aimed to prospectively evaluate the predictive power of ESS regarding the need for respiratory and/or renal support at discharge after emergent laparotomies; 1,347 patients fulfilled the inclusion criteria. On analysis, the authors concluded that ESS accurately predicted the need for respiratory and/or renal support at discharge. They suggested that ESS could be used for preoperative patient counseling and as a tool for quality-of-care benchmarking. In a post-hoc analysis of the EAST multicenter study, El Hechi et al. tried to find a correlation between ESS and mortality, morbidity, and the need for ICU admission for elderly patients undergoing emergency laparotomy [21]. This was assessed in three patient cohorts classified according to age, i.e., between 65-74, 75-84, and more than 85 years old. A total of 715 patients fulfilled the inclusion criteria. On analysis, the authors concluded that ESS predicted mortality, morbidity, and the need for ICU admission accurately in patients aged 65-74 years (C-statistic: 0.81, 0.75, 0.83 respectively). However, the predictive power of ESS was significantly lower in patients aged more than 85 years (C-statistic: 0.72, 0.64, 0.67 respectively).

AlSowaiegh et al. examined if ESS could predict the discharge of emergency surgical patients [22]. Based on the analysis of data from 84,694 patients, the authors found that based on a mean ESS of 5, the tool accurately and reliably predicted the discharge of the patients with a C-statistic of 0.83. They concluded that ESS can be used to predict discharge from hospital to home, a rehabilitation center, or other nursing facilities and thus could be used for preoperative counseling and planning discharge. Naar et al. hypothesized that ESS could accurately predict 30-day morbidity, mortality, and requirement for postoperative ICU care in patients undergoing emergency surgery, even with missing data variables [23]. Of the 359,849 who underwent emergency surgeries, 256,278 (71.2%) patients had at least one variable missing. They found that ESS correlated extremely well with mortality (C-statistic: 0.94), the need for postoperative ICU admission (C-statistic: 0.91), and morbidity (C-statistic: 0.77).

Discussion

Acute care surgery involves surgery for trauma, surgical critical care, and EGS [24]. Emergency surgery is defined as any surgical intervention that is essential to deal with an acute threat to life, organ, or tissue due to trauma, any progressive acute disease, acute exacerbation of the chronic disease, or a complication of a surgery or an interventional procedure [25]. Patients coming for EGS pose unique problems irrespective of their age and comorbidities. These include acute physiological derangements, lack of optimization, presence of chronic diseases, advanced age, underlying infections, immunosuppression, history of smoking, and alcoholism [26].

According to the American Association for the Surgery of Trauma (AAST), the ideal risk stratification tool should accurately quantify morbidity and mortality risk, use readily obtainable objective data, should be applicable early before surgical intervention and applicable in non-surgical cases as well, can be easily applied in clinical practice, and should be able to facilitate medical audits [27]. Although the time for optimization before an emergency surgery is usually less, Poulton et al. have recommended appropriate broad-spectrum antibiotics, adequate fluid replacement and electrolyte imbalance correction, management of ongoing medications, nutritional optimization, and glycemic control [28].

The presence of frailty in elderly patients undergoing emergency surgery is also responsible for postoperative mortality, complications, prolonged length of hospital stay, and the loss of independence after surgery [29]. Kennedy et al. suggested that frailty scoring should be incorporated into preoperative acute surgical assessment to help in decision-making and for developing novel postoperative strategies [30]. The Clinical Frailty Scale is a 9-point scale that is used to summarize the overall level of fitness or frailty of older adults when they undergo an evaluation by a healthcare professional. The higher the score, the greater the risk [31,32].

Enhanced Recovery After Surgery (ERAS) is a multimodal perioperative care pathway designed and validated to achieve early recovery for patients undergoing major surgery [33,34]. Although initially ERAS pathways were supposed to be feasible for elective surgeries only as the pathways are separate for preoperative, intraoperative, and postoperative phases, the pathways have been successfully used for emergency surgeries as well. Sharma et al. randomized 100 patients with acute small bowel obstruction or intestinal perforation and equally divided them into ERAS group and conventional pathways [35]. The authors concluded that length of hospital stay, surgical site infections, and chest infections were significantly lower in the ERAS group than in conventional pathways. The other advantages are an early return of bowel function, lower readmission rates, lower cost of treatment, lower mortality, and re-exploration [36].

There are many validated risk stratification tools currently available for use. However, most of the popular tools like APACHE II and P-POSSUM have significant limitations when they are used in patients undergoing emergency surgeries. Based on the review of published literature, ESS appears to be a comprehensive scoring tool that can reliably predict adverse perioperative outcomes in patients undergoing EGS.

This review has a few limitations. The review involves the prediction of 30-day postoperative events and not long-term outcomes following discharge. Most of the articles published have conclusions based on retrospective data gathered from national databases. There is a high possibility of missing patients with incomplete data. Most of the studies did not specify the type of surgeries and their numbers or percentages. This also leaves a large information gap. One of the variables assigned was belonging to the white race. It is not clear why there is a difference in the risk stratification if the patient belongs to a particular race, and this needs to be investigated further. Further prospective studies are required to ascertain the utility of ESS as a risk predicting tool when used prospectively.

## Conclusions

ESS is a simple risk assessment tool for predicting morbidity, mortality, and the need for ICU admission, predicting infectious complications like pneumonia and renal failure. This tool has been validated by several studies. ESS can also be used for patient counseling, prognostication of family members, and for benchmarking the quality of emergency surgical interventions. Further studies are needed to compare it with other risk scoring tools like P-POSSUM and APACHE II.
